# Fabrication, Evaluation, and Antioxidant Properties of Carrier-Free Curcumin Nanoparticles

**DOI:** 10.3390/molecules28031298

**Published:** 2023-01-29

**Authors:** Jinwei Wu, Jiaxin Chen, Zizhan Wei, Pingchuan Zhu, Bangda Li, Qing Qing, Huimin Chen, Weiying Lin, Jianyan Lin, Xuehui Hong, Fei Yu, Xiaodong Chen

**Affiliations:** 1 Medical College, Guangxi University, Nanning 530004, China; 2 The Fourth People’s Hospital of Nanning, Nanning 530023, China; 3 State Key Laboratory for Conservation and Utilization of Subtropical Agro-Bioresources, College of Life Science and Technology, Guangxi University, Nanning 530004, China; 4 Guangxi State Key Laboratory of Electrochemical Energy Materials, Guangxi University, Nanning 530004, China; 5 Department of Gastrointestinal Surgery, Zhongshan Hospital of Xiamen University, Xiamen 361005, China; 6 Suzhou Key Lab of Green Chemical Engineering, School of Chemical and Environmental Engineering, College of Chemistry, Chemical Engineering and Materials Science, Soochow University, Suzhou 215123, China

**Keywords:** curcumin, carrier-free, nanoparticles, antioxidant, food

## Abstract

Curcumin (Cur), a natural hydrophobic polyphenolic compound, exhibits multiple beneficial biological activities. However, low water solubility and relative instability hinder its application in food fields. In this study, carrier-free curcumin nanoparticles (CFC NPs) were prepared by adding the DMSO solution of Cur into DI water under continuous rapid stirring. The morphology of CFC NPs was a spherical shape with a diameter of 65.25 ± 2.09 nm (PDI = 0.229 ± 0.107), and the loading capacity (LC) of CFC NPs was as high as 96.68 ± 0.03%. The thermal property and crystallinity of CFC NPs were investigated by XRD. Furthermore, the CFC NPs significantly accelerated the release of Cur in vitro owing to its improved water dispersibility. Importantly, CFC NPs displayed significantly improved DPPH radical scavenging activity. Overall, all these results suggested that CFC NPs would be a promising vehicle to widen the applications of Cur in food fields.

## 1. Introduction

Curcumin (Cur), as a natural pigment, has been widely used as a spice in China and India [1]. Meanwhile, Cur exhibits a wide range of functional and biological features such as antioxidant and antidiabetic properties [2]. Despite its remarkable health benefits, Cur suffers from several limitations in the food and pharmaceutical industries due to its low solubility, poor stability, and rapid metabolism in the body [3]. In response to these problems, many Cur-loaded nanoparticle-based drug delivery systems (DDS) have been exploited in previous works including polymeric nanoparticles, gold nanoparticles, and mesoporous silica nanoparticles [4]. Nevertheless, most of the traditional carrier-assistant drug delivery systems require large quantities of additional excipients, which would induce low drug-loading efficiency [5]. In essence, the drug-loading efficiency of most proposed Cur-loaded nanosized drug delivery systems was less than 10%. Most of these inert carrier materials induce long-term toxicities because of their unclear metabolites [6]. In addition, the use of some carriers results in an undesirable immune response, which impaired the therapeutic effects. High drug-loading nanomedicines should be developed without the use of inert carrier materials [7]; furthermore, the application of many biopolymers (i.e., zein) in the food industry is restricted due to the toxicity of large amounts of organic reagents, as well as the extra cost of removing these organic reagents from the system [8]. It is important to minimize the use of organic solvents for making commercial food products nowadays [9].

Recently, carrier-free nanodrugs, constructed with amphiphilic drug–drug conjugates or pure drug molecules, have attracted extensive attention because of their easy-making method, excellent drug-loading capability, and the avoidance of large amounts of organic reagents [10]. As an emerging generation of DDS, carrier-free nanodrugs displayed many advantages: (i) excellent drug loading capacity (usually >90%); (ii) no carrier-induced immunogenicity and toxicity; (iii) avoiding any high energy-consuming machine in the preparation of carrier-free nanodrugs [11]; (iv) without using complicated procedures for preparing carriers; and (v) being suitable for most small chemical drug molecules. Zhao et al. reported the carrier-free 7-ethyl-10-hydroxycamptothecin/chlorin e6 nanoparticles by the self-assembly of pure drugs, which exhibited increased inhibition to cancer cells and realized an excellent antitumor effect in vitro [10]. Although this strategy has been widely applied in anti-tumor drugs, few nutraceutical-based carrier-free nanodrugs have been reported.

**Scheme 1:**
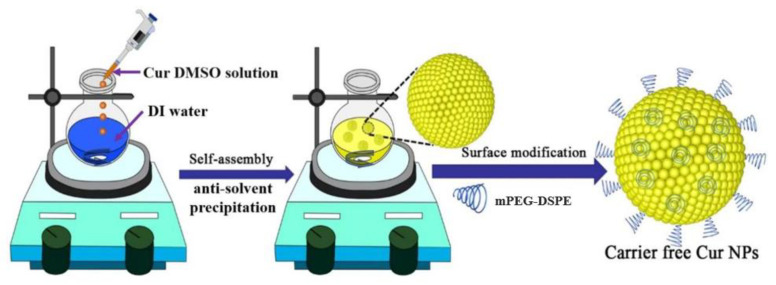
Schematic illustration of the CFC NPs.

In this study, CFC NPs were developed via the anti-solvent precipitation method (Scheme 1). During its self-assembly process, a tiny amount of mPEG-DSPE was used for the surface modification of pure CFC NPs [12]. The surface charge, mean particle size, and loading capacity of CFC NPs were systematically investigated. The physicochemical properties of the CFC NPs were also assessed by XRD, UV-Vis spectrum, TEM, SEM, and fluorescence spectrum. Furthermore, in vitro release profiles of Cur in simulated gastrointestinal fluids and the antioxidant activity of the CFC NPs were carefully evaluated [13].

## 2. Results and Discussion

### 2.1. Preparation and Characterization of CFC NPs

The CFC NPs were prepared by the anti-solvent precipitation method, which was displayed in Scheme 1. Firstly, the organic solvent (DMSO) was used to dissolve the Cur. Then, the DMSO solution of Cur was dropped into the DI water (non-solvent for Cur) with vigorous stirring. It should be noted that the supersaturated conditions of the Cur in the water phase would induce the precipitation or nucleation of the CFC NPs. Eventually, the Cur molecules self-assembled into nanoparticles with the driving forces of various noncovalent interactions. Furthermore, the amphiphilic stabilizer (mPEG-DSPE) was added to overcome the agglomeration of CFC NPs due to the supersaturation.

**Figure 1:**
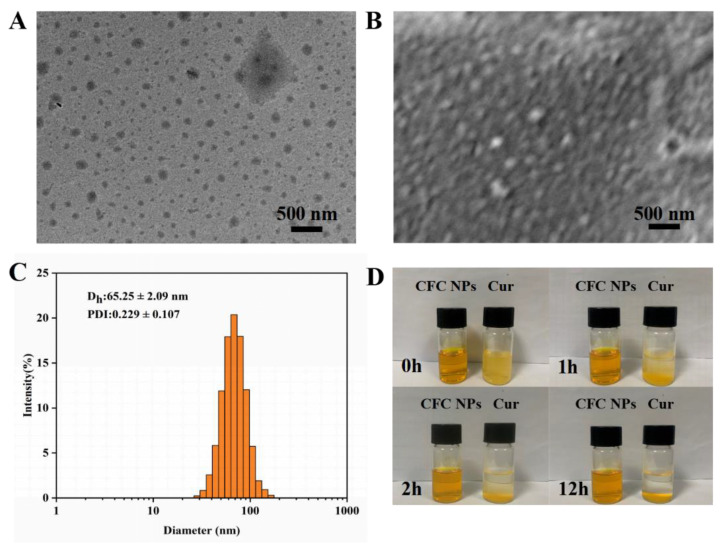
(**A**) TEM image and (**B**) SEM image of CFC NPs. (**C**) Particle size distribution and PDI of CFC NPs. (**D**) Visual appearances of CFC NPs and free Cur during 12 h of storage time.

As shown in Figure 1A,B, TEM and SEM indicated that the morphology of CFC NPs was nearly spherical, and the size of the morphological observation was basically consistent with the DLS results. In addition, CFC NPs were well dispersed on the support film, which might be ascribed to enhanced surface hydrophilicity after the addition of mPEG-DSPE. Furthermore, the obvious advantage of carrier-free nanodrug delivery systems is their high drug-loading capacities, usually ranging from 50% to 100% [14]. In this work, the loading capacity of CFC NPs was 96.68 ± 0.03%. As shown in Figure 1C, the average size of CFC NPs was 65.25 ± 2.09 nm with a narrow particle size distribution (0.229 ± 0.107), which proved the uniform property of CFC NPs [15]. Meanwhile, the zeta potential (−20.5 ± 1.3 mV) of CFC NPs was high enough to prevent aggregation of CFC NPs [16].

Storage stability is important for the practical application of CFC NPs in the food industry. The visual appearance of CFC NPs during 12 h of storage time at room temperature was presented in Figure 1D. The aggregates of Cur were formed within 1 h because of their low water solubility of 11 ng/mL [17]. In contrast, the CFC NPs were preserved in a transparent state without obvious precipitation over time, which indicated that CFC NPs were stable under storage conditions. These results might be attributed to the fact that the hydrophilic surface increased the water dispersibility of Cur, and the negative charge of CFC NPs provided the electrostatic repulsion to inhibit the aggregation [18].

### 2.2. UV-Vis and Fluorescence Spectroscopy Analysis

**Figure 2:**
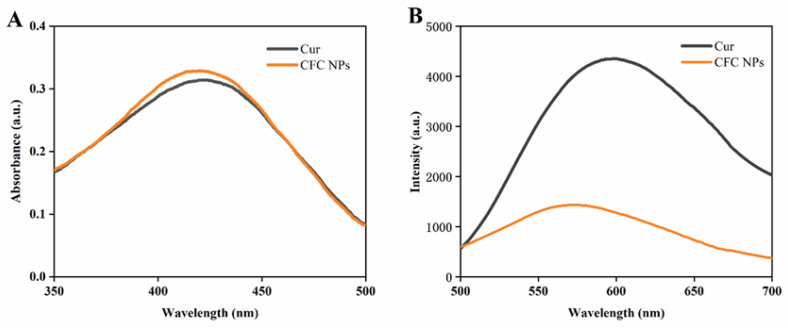
(**A**) UV-Vis absorption spectra and (**B**) fluorescence spectra of free Cur and CFC NPs.

To further investigate the spectral changes of the CFC NPs, the UV-Vis and fluorescence spectra of free Cur and CFC NPs were analyzed. As shown in Figure 2A, the UV-Vis spectrum of CFC NPs was somewhat different from that of free Cur [19]. The spectrum of CFC NPs showed a slight blue shift, and the maximum absorption spectra of Cur shifted from 423 to 420 nm. A similar behavior was observed in the fluorescence spectra (Figure 2B), where the emission maxima shifted from 595 to 585 nm. The blue shift of the Cur peak could be the result of noncovalent interactions in CFC NPs, such as π-π stacking interactions [20]. Notably, the fluorescence intensity of CFC NPs was dramatically decreased compared to that of free Cur, which declared that the collision and aggregation among the CFC NPs seemed to result in fluorescence quenching. These results preliminarily confirmed that CFC NPs were successfully fabricated [21].

### 2.3. XRD Analysis

**Figure 3:**
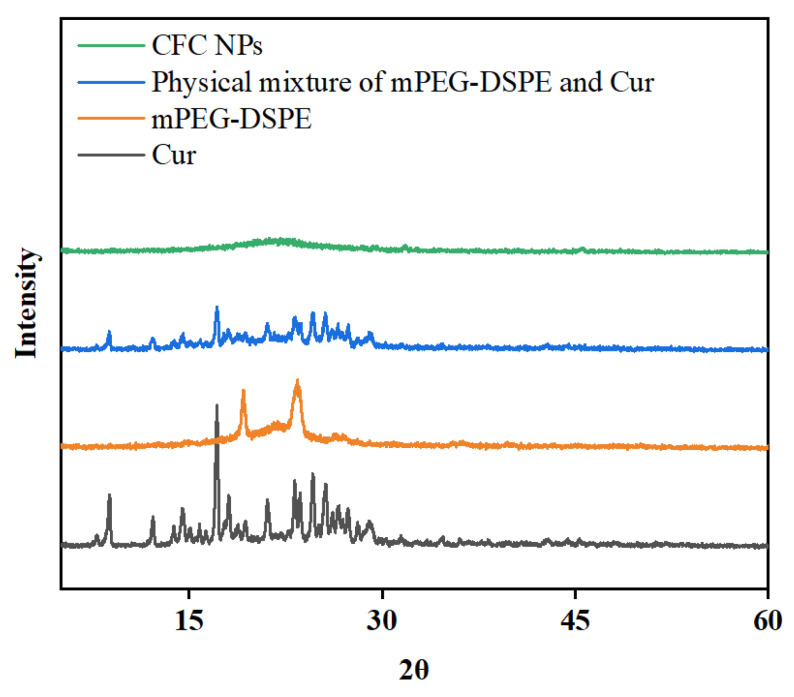
X-ray diffraction patterns of CFC NPs, physical mixture of mPEG-DSPE and Cur, mPEG-DSPE, and Cur, respectively.

As shown in Figure 3, XRD was carried out on Cur, mPEG-DSPE, a physical mixture of mPEG-DSPE and Cur, and CFC NPs to ascertain the physical state of Cur within CFC NPs. Free Cur was in a highly crystallized state and exhibited many sharp peaks at 8.78°, 12.20°, 14.42°, 17.14°, 23.18°, 24.64°, 25.62°, and 29.04°, which was consistent with previous reports [22]. It should be noted that CFC NPs did not show these sharp characteristic peaks of Cur, which indicates that Cur changed from a crystalline state into an amorphous state after being encapsulated in CFC NPs. In contrast, these diffraction peaks of Cur still existed in the physical mixture. This indicates that the formation of CFC NPs indeed changed the crystalline state of Cur. These results indicated that once encapsulated in CFC NPs, Cur existed in an amorphous state.

### 2.4. Photostability Assay

**Figure 4:**
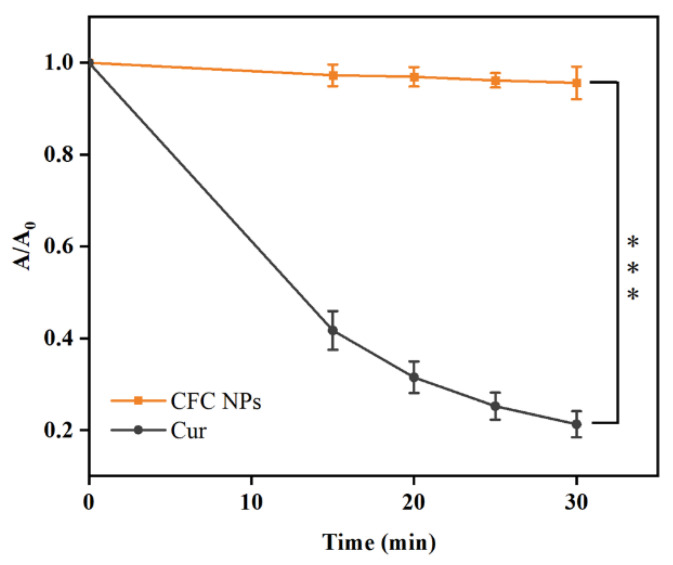
Photo-decomposition profiles of CFC NPs and free Cur after UV-light irradiation for 30 min. *** *p* < 0.001.

Interlinked 2-hydroxymethoxy benzene rings exist in Cur molecules, which are unstable when exposed to light. Indeed, photochemical degradation is one of the reasons for their loss of biological activity. As demonstrated in Figure 4, the free Cur showed a rapid degradation rate. About 60% of Cur molecules were degraded after 15 min, and only 21% survived after 30 min. In comparison, the CFC NPs showed a retardation phenomenon, and only 5% of Cur molecules were degraded after 30 min light treatment. The observations clearly indicated that the CFC NPs significantly reduced the degradation rate of Cur and provided better protection under UV light exposure. This would be conducive to the utilization of Cur in the field of functional foods.

### 2.5. In Vitro Release Study

**Figure 5:**
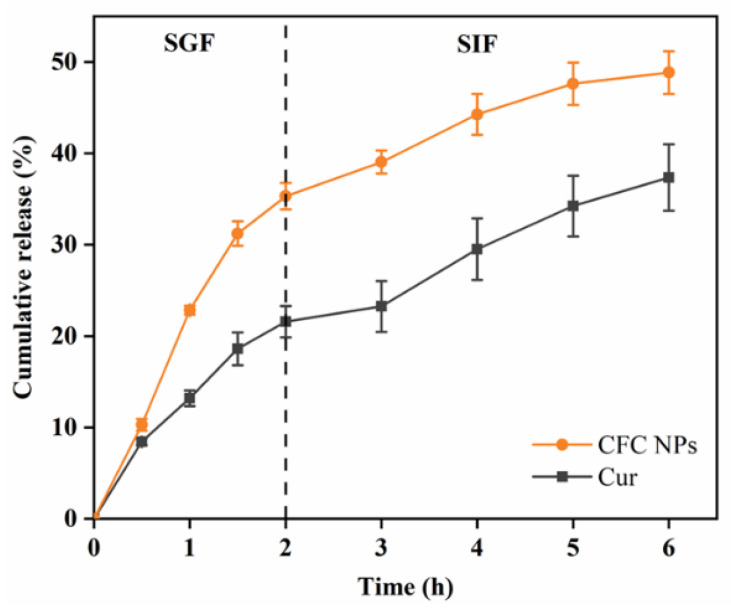
In vitro drug release profiles of CFC NPs and free Cur. SGF and SIF represent simulated gastric fluid and simulated intestinal fluid, respectively.

The drug release profiles of CFC NPs were shown in Figure 5. Free Cur and CFC NPs exhibited a similar release trend in the SGF and SIF, which referred to a rapid release in SGF and then a relatively slower release in the SIF condition. A burst release occurred in CFC NPs at the beginning of SGF, which was probably related to the dissolution of Cur on the surface of NPs. After 2 h of incubation in SGF, the drug release of CFC NPs was ~31.31%. In contrast, free Cur released at a slow rate (~21.58%) owing to its poor solubility in the gastrointestinal fluid. When the samples were transferred from SGF to SIF, a sustained release of Cur could be clearly observed. After 4 h of incubation in SIF, the cumulative release rate of free Cur and CFC NPs was ~37.35% and ~48.84%, respectively. The CFC NPs showed higher drug release in the SGF and SIF, compared to that of free Cur. This phenomenon is attributed to the larger surface area and the enhanced dispersibility in water. This similar phenomenon was also reported by Xiao et al. [23]. The above results indicated that the formation of CFC NPs successfully promoted the release of Cur in simulated digestive juices, which would allow more Cur to be absorbed by the body.

### 2.6. Antioxidant Activity

**Figure 6:**
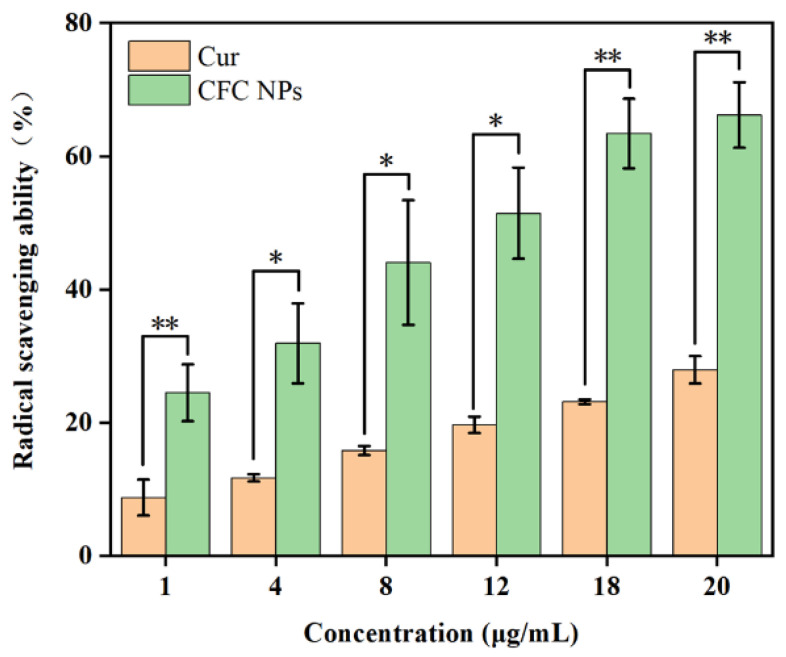
DPPH radical scavenging activity of CFC NPs and free Cur. * *p* < 0.05, ** *p* < 0.01.

Cur, a phenolic compound, has been evidenced to exhibit antioxidant activity through forming phenoxy groups to remove free radicals [24]. The DPPH radical-scavenging method was broadly employed to evaluate antioxidant capacity owing to its cost-effectiveness, high sensitivity, and good reproducibility [25]. As shown in Figure 6, both the CFC NPs and free Cur displayed dose-dependent DPPH radical scavenging activity. At the concentration of 20 μg/mL, the DPPH radical scavenging activities of free Cur and CFC NPs were ~27.95% and ~66.18%, respectively. These results indicated that CFC NPs exhibited a remarkably higher antioxidant activity than that of free Cur. This was attributed to the Cur being encapsulated in CFC NPs with a hydrophilic surface, which provided better contact between Cur and free radicals. Several other studies also reported this similar phenomenon [26,27,28]. These findings imply that the CFC NPs could be used as antioxidant ingredients in food formulations, which would be beneficial for the human body.

## 3. Materials and Methods

### 3.1. Materials

Curcumin (purity > 97%) was acquired from Dalian Meilun Biotechnology Co., Ltd. (Dalian, China). 1,2-Distearoyl-sn-glycero-3-phosphoethanolamine-*N*-methoxy-poly (ethylene glycol 2000) (mPEG-DSPE) was purchased from Shanghai ToYongBio Tech. Inc., Ltd. (Shanghai, China). All other solvents including dimethyl sulfoxide (DMSO) and ethanol were provided by Sinopharm Chemical Reagent Co., Ltd. (Shanghai, China).

### 3.2. Preparation of CFC NPs

Briefly, 2 mg of Cur was dissolved in 1 mL of DMSO. Then, 250 μL of the above solution was injected dropwise into 10 mL of DI water under stirring (40 °C). Subsequently, 50 μg of mPEG-DSPE was added. Then, the CFC NPs were finally formed. The DMSO was removed by the dialysis method for the evaluation of subsequent experiments.

### 3.3. Characterization of CFC NPs

The particle size distribution of the newly prepared CFC NPs was measured using dynamic light scattering (Zetasizer Nano-ZS90, Malvern Instruments, Worcestershire, UK). Experimentally, each sample was diluted with deionized water for analysis, and then some of the diluted solution was filtered by a 0.45 μm PES (polyether sulfone) membrane. After filtering, the hydrodynamic diameter and PDI of CFC NPs were measured at 25 °C, and each value was measured at least three times.

Morphology of the formed CFC NPs was investigated with a TEM. A copper grid (400 mesh) was used to carry 200 μL of the sample. The CFC NPs were observed by TEM after drying for 12 h at 25 °C. In addition, the surface morphology of the obtained CFC NPs was observed using an SEM (Hitachi, Tokyo, Japan). CFC NPs were dried on silicon pellet at room temperature. The samples were sputter coated with gold before imaging.

The samples of Cur and freshly prepared CFC NPs were placed in a glass bottle at room temperature. The appearance of samples was recorded at predetermined time intervals (0, 1, 2, and 12 h).

### 3.4. Loading Capacity (LC)

Loading capacity was calculated after determining the concentration of Cur by UV-Vis spectroscopic method. The CFC NPs were firstly centrifuged at 1000× *g* for 10 min. Then, the precipitation resulting from the centrifugation was removed. Liquid supernatant was dissolved using DMSO-water solution (DMSO:H_2_O = 4:1). The above sample was quantified using UV-Vis spectrophotometer at 425 nm. In addition, a Cur calibration curve at different concentrations was obtained: Y = 0.1504X + 0.0164 (R^2^ = 0.9994), where Y and X refer to the absorbance and concentration of Cur, respectively. The loading capacity (LC) was calculated by the following equation:

LC (%) = (total Cur − free Cur)/total amount of nanoparticles × 100

### 3.5. Spectroscopic Techniques

The UV-Vis absorption spectra of samples were measured in the wavelength range of 350 to 500 nm. The corresponding solution was used for blank correction.

The fluorescence of free Cur and CFC NPs was analyzed via a fluorescence spectrophotometer (Synergy H1, BioTek Instruments, Inc., Winooski, VT, USA). The excitation wavelength of all samples was recorded at 442 nm.

### 3.6. XRD Patterns

The XRD patterns of free Cur, physical mixture of mPEG-DSPE and Cur, mPEG-DSPE, and lyophilized CFC NPs powders were recorded on X-ray diffractometer (Rigaku D/MAX 2500V). The CFC NPs were pre-frozen at −60 °C for 4 h and freeze-dried to obtain the powders of CFC NPs. The obtained samples were determined from 5° to 60° at a speed of 10°/min.

### 3.7. Photostability Assay

Freshly prepared samples of free Cur and CFC NPs were exposed to UV light (254 nm). Then, the samples were irradiated for a certain time (0, 15, 20, 25, and 30 min). The retention rate of Cur was calculated:

Retention rate of Cur = A/A_0_

where A and A_0_ represent the absorbance at different time points and the initial absorbance of Cur, respectively.

### 3.8. In Vitro Simulated Gastrointestinal Studies

The in vitro release of the free Cur and CFC NPs was analyzed using the dialysis bag method. Briefly, the release medium for Cur was obtained by adding SGF or SIF into ethanol. Then, 6 mL of CFC NPs or free Cur solution was added into the dialysis bags (MWCO of 8000–14,000 Da). They were submerged in 100 mL of SGF release medium for 2 h at 37 °C. After that, the dialysis bags were transferred to 100 mL SIF release medium and kept for 4 h at 37 °C. At specific times, 4 mL of sample was collected and replaced with 4 mL of fresh release medium into flask. The percentage of the released drug was obtained by determining the Cur content of the above-collected samples.

### 3.9. Antioxidant Activity

The antioxidant activity of the free Cur and CFC NPs was examined by the method previously described [29]. Initially, DPPH ethanol solution and multiple concentrations of free Cur and CFC NPs were prepared. Then, 1 mL of DPPH ethanol solution was mixed with of sample solution and stored at 25 °C for 30 min in dark condition. Finally, a microplate reader analyzed the absorbance of samples (at 517 nm). Blank solution was also prepared by mixing DPPH ethanol solution with water, and DPPH radical scavenging activity was estimated using the previously reported formula [30,31].

### 3.10. Statistical Analysis

All measurements were repeated at least three times. Data were recorded as mean ± standard deviation and statistically analyzed for a significant difference by GraphPad Prism 9.3.0.

## 4. Conclusions

In the present work, CFC NPs with a high drug loading capacity (96.68 ± 0.03%) were successfully fabricated. Various noncovalent interactions might be the dominant driving forces for facilitating the formation of CFC NPs. With the functionalization of biocompatible mPEG-DSPE, the CFC NPs showed excellent water dispersibility and improved antioxidant activity. Meanwhile, the CFC NPs exhibited a small particle size of 65.25 ± 2.09 nm, a zeta potential of −20.5 ± 1.3 mV, and a spherical shape. Overall, this study provides an effective approach to deliver nutraceuticals, such as Cur, which could be applied in the functional food and pharmaceutical industries.

## Data Availability

The data presented in this study are available from the corresponding author upon request.

## Sample Availability

Not available.
